# Strategies to reduce risks in ARV supply chains in the developing world

**DOI:** 10.9745/GHSP-D-14-00105

**Published:** 2014-12-02

**Authors:** Chris Larson, Robert Burn, Anja Minnick-Sakal, Meaghan O'Keefe Douglas, Joel Kuritsky

**Affiliations:** a UPS, Columbus, Ohio, USA; b Management Sciences for Health, Arlington, VA, USA; c Formerly with the United States Agency for International Development, Washington, DC, USA; d United States Agency for International Development, Global Health Fellows Program, Washington, DC, USA; e United States Agency for International Development, Washington, DC, USA

## Abstract

Key strategies of the main ARV procurement program for PEPFAR to reduce supply chain risks include: (1) employing pooled procurement to reduce procurement and shipping costs and to accommodate changing country needs by making stock adjustments at the regional level, and (2) establishing regional distribution centers to facilitate faster turnaround of orders within defined catchment areas.

## BACKGROUND

Between September 2005, when the United States Agency for International Development (USAID) awarded the Supply Chain Management System (SCMS) project, and August 2014, the SCMS project delivered over US$1.9 billion in HIV/AIDS commodities, approximately $1.1 billion of which were antiretroviral (ARV) drugs to support the treatment of people with HIV and AIDS. This total sum of ARVs accounted for approximately two-thirds of the ARVs delivered with funding from the US President's Emergency Plan for AIDS Relief (PEPFAR).^1^ The SCMS project is led by the Partnership for Supply Chain Management, with 13 private-sector and nongovernmental partners.^2^

One of the primary challenges to achieving a reliable, cost-effective, and secure supply chain of HIV/AIDS commodities has been the high cost of commodities. The US Department of Health and Human Services and the US Food and Drug Administration (FDA) made a significant initial response to the issue of cost in May 2004, when they introduced a new initiative to facilitate global ARV manufacturers' access to an expedited product review and inspection process. This tentative approval process to determine the quality of ARVs produced by manufacturers anywhere in the world was designed to increase access to high-quality medications globally. Under this initiative, if products meet the FDA's quality assurance standards, they are eligible for purchase under PEPFAR.^3^

As generic products obtained approval for use under PEPFAR, stakeholders began to procure more generic ARVs and fewer branded ones, resulting in rapid and substantial savings to PEPFAR. A 2010 study estimated that **between 2005 and 2008 PEPFAR saved over US$323 million by procuring approved generic ARVs instead of equivalent-branded ARVs**.^4^

Between 2005 and 2008, PEPFAR saved over an estimated US$323 million by procuring approved generic ARVs instead of branded ones.

As of September 30, 2013, 6.7 million people were receiving antiretroviral treatment through the PEPFAR program—up fourfold from 1.7 million in 2008.^5^ Addressing demand-side challenges while simultaneously mitigating supply risks (production and shipping delays) as well as cost risks has been essential to meeting the needs of antiretroviral therapy (ART) programs and ensuring that a stable supply of ARVs is available to patients.

This article describes the practices employed by USAID and the SCMS program to mitigate ARV supply chain risks. From the program's experience in numerous PEPFAR countries, the risks can be grouped into 3 categories of supply, demand, and cost (Table).

**TABLE.** ARV Supply Chain Risks and Corresponding Risk Mitigation Strategies by the SCMS Project

**Risk Category**	**ARV Supply Chain Risks**	**Risk Mitigation Strategies**
Supply	• Production delays	• Multiple-source procurement
	• Shipping delays	• Pooled procurement
		• Use of RDCs
		• Flexible product specification
Demand	• Expanding treatment programs	• Frequent update and review of supply plans
	• Inaccurate and/or delayed demand forecasting and supply planning	• Regional aggregation of country forecasts and supply plans for pooled procurement
	• Burdensome procurement procedures	• Restocking RDCs based on analysis of likely demand (as opposed to firm orders)
Cost	• High per-unit product costs	• Pooled procurement based on aggregate demand plans
	• High shipping costs	• Freight consolidation and ocean shipping

Abbreviations: ARV, antiretroviral; RDC, regional distribution center; SCMS, Supply Chain Management System.

The program's risk mitigation approaches represent integrated supply chain strategies that take advantage of economies of scale to improve cost and quality. In particular, **pooled procurement and the use of regional distribution centers (RDCs)** are common strategies across the 3 risk categories, illustrating the interconnectedness of risks. For example, inaccurate forecasting (demand risk) may lead to under-procurement (supply risk), eventually resulting in the need for a costly emergency order (cost risk) to remedy a product stock-out.

## SUPPLY RISKS

Production and shipping delays can result in product stock-outs when goods are not delivered according to the supply plan and, hence, are not available for scheduled in-country distribution. These stock-outs can lead to ART interruption for patients if the needed ARV drugs are not consistently available. Country-specific procurements are scheduled according to a multilateral, agreed-upon supply plan, designed to maintain inventory levels across the in-country supply chain.

According to SCMS procurement data, between 2007 and 2012, manufacturers of generic ARVs delivered their products on time to the contractually agreed-upon destination (the RDC or a country's central medical store) 50% to 70% of the time. Orders were considered on time if they arrived at their destination within 14 days of the projected delivery date in the price quote. Of the orders arriving late (greater than 14 days after the projected delivery date), two-thirds were provided within 1 month of the originally agreed-upon delivery date.^6^ In order to improve the delivery performance due to production or shipping delays to the recipient countries the program developed the following strategies to mitigate supply risks.

These 4 strategies are:

1. Multiple-source procurement
2. Pooled procurement based on projected demand
3. Use of RDCs
4. Flexible specifications for presentation of ARVs (appearance of packaging, quantities per package, instructions printed in multiple languages) to facilitate common product use across multiple countries

### 1. Multiple-Source Procurement Mitigates Production and Shipping Delays

Based on the best-value approach that is embodied in USAID procurement regulations, ARV procurements are usually awarded concurrently to multiple vendors on a tender-by-tender basis. The benefits of doing so are twofold. First, the risk and consequence of sudden production or delivery failures by a single vendor are mitigated by the completion of orders for the same product by other vendors. Second, it is the opinion of the authors that the participation of multiple vendors leads to an ARV marketplace that is competitive and growing in capacity to supply these products to an increasing number of patients on ART. Starting in 2009, the program began to issue the vast majority of its tenders as multiple-source tenders.

### 2. Pooled Procurement Maximizes Product Availability and Reduces Costs

Pooling (combining) the procurement for common ARVs across PEPFAR-supported countries based on their supply plans maximizes the availability of these ARVs and reduces procurement and shipping costs for a number of reasons:

- Economies of scale are attained for manufacturing and shipping.
- Fewer tenders and orders need to be processed, reducing transactional costs for both the buyer and the manufacturers.
- Minimum batch volumes imposed by manufacturers before beginning production are reached more rapidly, thus avoiding production delays.
- The capacity of shipping containers can be maximized fully, particularly when pooled products are shipped to RDCs.

Pooled procurement among neighboring countries reduces procurement and shipping costs.

The pooled procurement process also allows vendors and recipients to plan ahead strategically. For example, when countries place orders at least 6 months before they need the goods, ocean freight can be used rather than more expensive air freight. Additionally, by pooling procurement, SCMS can negotiate reduced aggregate tendering rates. The program issues approximately 8 planned tenders to the market per year, of large aggregate volumes each time. This process increases the program's negotiating power with vendors, improving the chances of obtaining the best price available, in contrast to an annual tender process, which allows only for a single negotiation. Using a continuous retendering and buying strategy throughout the year also allows the program to take advantage of price declines in the market.

Using a continuous retendering strategy throughout the year for large aggregate volumes increases the program's negotiating power and allows it to take advantage of price declines in the market.

By combining demand forecasts with procurement history from multiple countries (normally within the same region), SCMS can hedge against forecast errors. The combined forecast is shared quarterly with manufacturers, suppliers, other implementing partners, and nongovernmental organizations, such as the Global Fund to Fight AIDS, Tuberculosis, and Malaria, the Clinton Health Access Initiative, and the United Nations Children's Fund (UNICEF).

**Figure 1.**
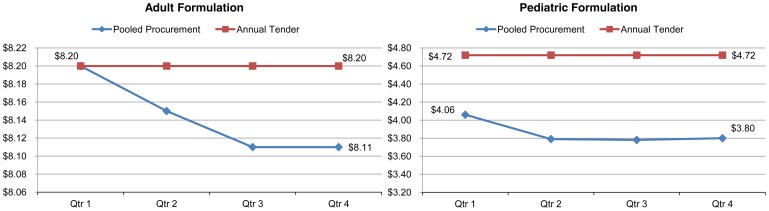
2013 Pricing (US$) for Adult and Pediatric Formulations of Fixed-Dose Combination Antiretroviral Drugs,^a^ Pooled Procurement vs Annual Tender^b^ ^a^ Adult formulation: Lamivudine/Nevirapine/Zidovudine 150/200/300 mg dispensed in 60 tabs; pediatric formulation: Lamivudine/Nevirapine/Zidovudine 30/50/60 mg dispensed in 60 tabs. ^b^ Pooled procurement across PEPFAR-supported countries; annual tender in an East African country.

To illustrate the cost savings from the pooled procurement model, the authors have prepared the comparison below between the 2013 annual tender process in an East African country and the pooled procurement price in 2013 for two high-volume fixed-dose combination (FDC) ARVs (Figure 1). The pooled procurement price for the adult formulation of Lamivudine/Nevirapine/Zidovudine (150/200/300 mg dispensed in 60 tabs) and for the pediatric equivalent (30/50/60 mg dispensed in 60 tabs) trended below the East African country's annual tender price, with modest differences at the start and a widening gap throughout the year 2013. This is an illustrative example of the potential benefit of frequent tendering, founded on pooled procurement, which enables the buyer to capture price decreases throughout the year.

### 3. Use of Regional Distribution Centers Speeds the Order-to-Delivery Process

**Figure 2.**
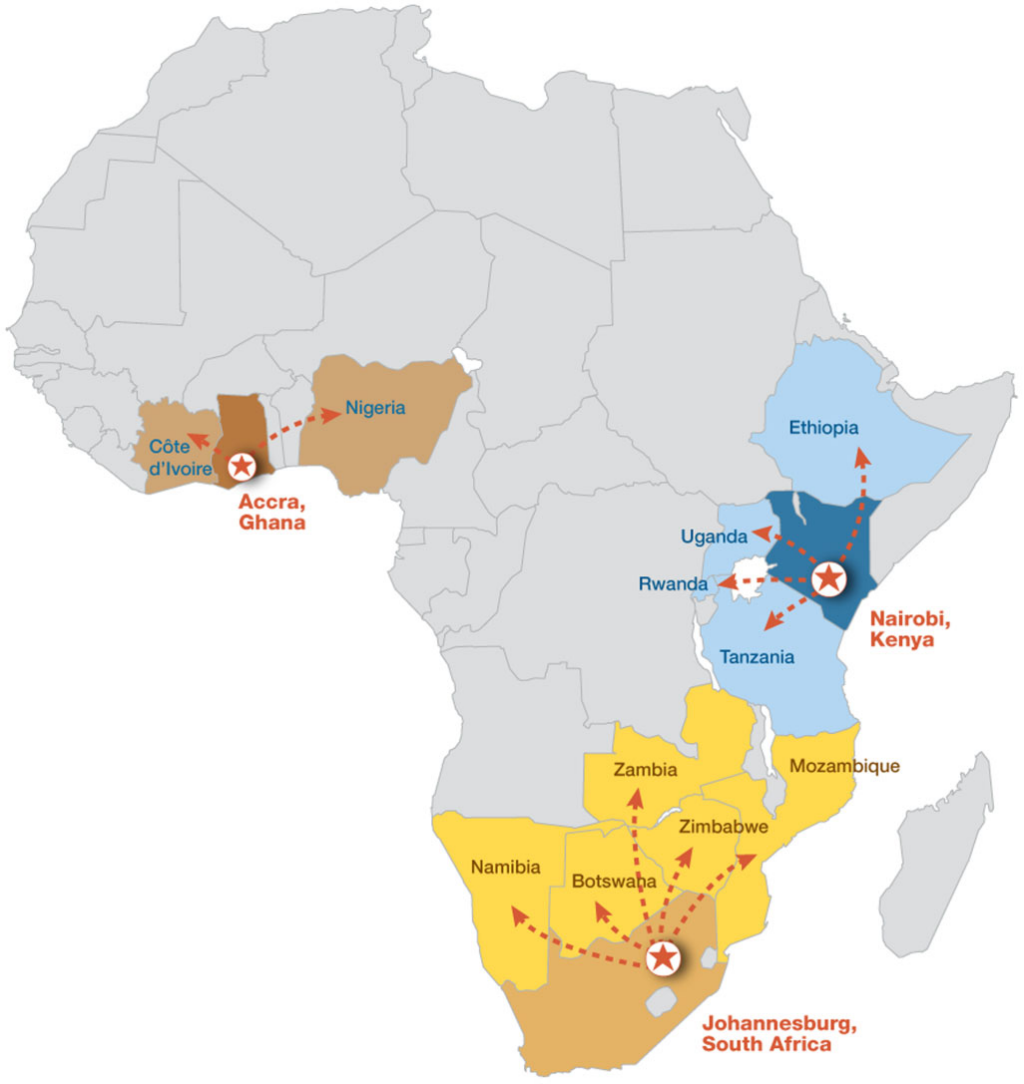
Catchment Area of Regional Distribution Centers To support global pooled procurement and reduce turnaround times for delivery of ARVs, the Supply Chain Management System project established 3 regional distribution centers (RDCs) in Ghana, Kenya, and South Africa. These facilities hold strategic stock, provide regularly scheduled shipments to neighboring countries, and expedite emergency orders to prevent stock-outs. To provide a sustainable resource, the RDCs were established as independent commercial enterprises, attracting major private-sector pharmaceutical clients.

The program has established 3 RDCs to provide intermediate warehousing and regional distribution for 11 other PEPFAR-supported countries in sub-Saharan Africa (Figure 2). For example, the RDC in Pretoria, South Africa, serves Botswana, Mozambique, Namibia, Zambia, and Zimbabwe. The 3 distribution centers, in turn, have supplied 40 countries with HIV/AIDS commodities and have enabled the program to effectively increase the availability and timely shipment of generic ARVs.

**Figure 3.**
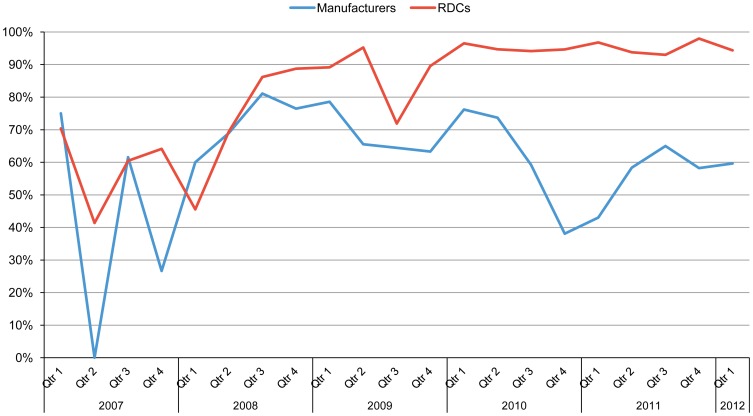
Proportion of ARV Shipments Delivered On Time, Manufacturers Versus SCMS Regional Distribution Centers, January 2007–March 2012 Abbreviations: ARV, antiretroviral; RDC, regional distribution center; SCMS, Supply Chain Management System.

One strategy for operating efficient RDCs involves holding common, high-volume generic ARVs in multimarket, pharmaceutical-grade distribution centers to facilitate more rapid turnaround of orders originating within the defined catchment region, reducing delivery time and improving on-time delivery to clients. This model has been used in the private sector as well.^7^ Between 2007 and 2012, the RDCs delivered 90% of orders on time. In contrast, between 50% and 70% of orders arrived on time with direct delivery from a vendor during the same time period (Figure 3).^6^ The RDCs are independent, commercial enterprises, which serve commercial and public sectors, ensuring their sustainability. The costs associated with RDC warehousing services have averaged 2.89% of the value of the ARVs delivered.

Between 2007 and 2012, 90% of orders fulfilled by regional distribution centers were delivered on time compared with only 50%–70% of orders fulfilled directly by vendors.

RDCs can also be leveraged to substantially reduce lead time–the time interval between the order and receipt of commodities. Analysis conducted in 2011 showed that partner country programs requesting emergency shipments (ie, shipments with a requested delivery date of less than 60 days from the order date) received delivery in an average of 28 days from RDCs, out of RDC stock, compared with an average of 72 days directly from manufacturers.^6^ This means that these intermediate storage facilities not only have sped up delivery but also have fulfilled urgent orders from stock positions when the minimum time frame for delivery could not otherwise have been met by manufacturers.

Regional distribution centers can be leveraged to substantially reduce lead time.

By managing the inventory of ARVs strategically at the RDCs, the program has been able to mitigate many supply and demand risks. Many factors are considered to ensure sufficient RDC stock levels. First, we anticipate demand from countries, which is determined by the ARV supply plans developed by each country as an outcome of ARV commodity forecasts.^8^ Supply plans also consider factors related to attributes of the market place for generic ARVs, such as lead time, manufacturer delivery performance, historical demand, the need to make stock available for emergency requirements, and product transitions due to revisions to treatment guidelines. Finally, supply plans are also informed by market dynamics issues, such as active pharmaceutical ingredient shortages, formulation production capacity constraints, new product entrants, and increasing options for fixed-dose combinations.

### 4. Flexible Specifications Facilitate Product Use in Multiple Countries

The program encourages manufacturers to register a common presentation for a given ARV formulation across multiple countries whenever possible, which allows the program to procure and ship a single product to most African countries. In the majority of cases, the single presentation uses labels and inserts printed in both English and French. This common presentation strategy has come to dominate the ARV procurements made by the program; in 2013, 77% of the ARVs procured by the program by volume were of the common presentation with English/French labeling.

## DEMAND RISKS

The authors define demand risks as operational matters at the client, program, or country level that impact the timely and cost-effective processing of ARV orders through the national and donor systems.

Producing accurate national demand forecasts is challenging, which can result in orders that may not reflect the country's actual need. On the one hand, under-forecasting can result in product scarcity, increasing the likelihood of stock-outs and creating the need for costly emergency orders. It also substantially increases the use of high-cost air shipping—the normal transportation mode for emergency orders. In contrast, orders placed sufficiently in advance of their desired delivery date can be shipped via ocean, with savings of more than 60% when compared with the cost of air freight. To ensure on-time delivery of the most common fixed-dosed combination and single-dose formulation ARVs shipped via ocean, a minimum lead time of 6 months is needed. This lead time can extend up to 9 months, as supply constraints and demand variations come into play.

Conversely, over-forecasting can result in excess stocks that expire before use. This increases costs of storage and handling as well as the cost of properly disposing of the unused product. Additional common challenges to timely, accurate procurement include delays in finalizing and submitting orders and coordination issues between donors and recipients.^8^

To mitigate these demand risks, the program has employed a combination of strategies:

1. Regularly updating country forecasts and supply plans
2. Regionally aggregating supply plans for pooled procurement (noted above as a measure that also maximizes supply-side product availability)
3. Restocking RDCs based on analysis of likely demand, rather than on firm orders from clients

### 1. Frequently Updated Country Supply Plans Improve Orders and Reduce Stock Imbalances

The program assists PEPFAR-supported countries in establishing 12–18 month supply plans, which are updated every 3 months and are based on reported product consumption. Delivery quantities for planned shipments can thus be continuously revised to reflect a more accurate picture of demand and to respond to changes in consumption trends, ultimately attempting to avoid both stock-outs and overstocking of commodities.

### 2. Aggregating Supply Plans for Pooled Procurement Enables Stock Adjustments at the Regional Level

Taking the additional step of aggregating the supply plans of all countries within a region further mitigates supply risks linked to forecasting weaknesses, because adjustments can be made closer to the country program, as opposed to the very beginning of the supply chain. For example, if one country in a region adjusted its supply plans to reflect greater ARV requirements and another country in the same region concurrently experiences reduced demand, the relevant RDC could transfer excess from the latter to meet the increased needs of the former without ever having to order new supplies from manufacturers.

### 3. Restocking Regional Distribution Centers Based on Likely Demand Allows for Flexibility at the Country Level

The program also uses aggregated supply plans to plan advanced ARV procurements delivered to the RDC in bulk shipments using ocean freight, which saves significant funds. The quantities from these shipments can then be broken down for distribution to countries in the same regions as the RDC. The RDC also allows for flexibility with clients by increasing or reducing delivery quantities of a product to a given country each quarter to align with changes in actual consumption where that data are available. Private-sector companies practice this sort of strategic inventory management by holding a certain amount of stock depending on the forecast and lead time associated with each product or component pieces, enabling the companies to fill orders quickly. ^9^

## COST RISKS

Cost risks may generally be divided into risks related to the unit cost of ARVs and risks related to shipping and storage. In short, efforts described above to reduce supply and demand risks through regionally aggregated planning, procurement, and intermediate storage also have a considerable effect on reducing costs throughout the supply chain.^10^

### 1. Pooled, Frequent Procurement Lowers Product Units Costs

Economies of scale obtained through pooled procurement create the opportunity for cost savings in the ARV supply chain.^10^^,^^11^ However, this is not only the result of reducing the number of demand-side transactions required to obtain the same quantity of products for multiple countries. Economies of scale are also achieved on the supply side, as manufacturers are asked to respond to fewer requests for proposals overall, since the program covers multiple countries in a single order. This reduces costly workloads associated with preparing separate outbound orders, export customs entries, transaction records, and invoices.

Pooled procurement provides an opportunity for cost savings through economies of scale.

### 2. Freight Consolidation Reduces Shipping Costs

The strategy of the program for transporting ARVs focuses on meeting client country requirements at the lowest possible cost without compromising product integrity. This means balancing the use of ocean and air freight for manufacturer-to-country shipments. Air freight is the best choice when urgency is needed to avoid stock-outs, treatment interruptions, and significant program disruptions; otherwise, optimizing available lead time for orders allows for lower-cost shipment by ocean, which the program seeks whenever possible. In addition, road freight rather than air can be used on key lanes for intra-Africa movements. The program strives to consolidate as many orders as possible into as few shipments as practical, leveraging the process to facilitate lower-cost modes of transport.

**Figure 4.**
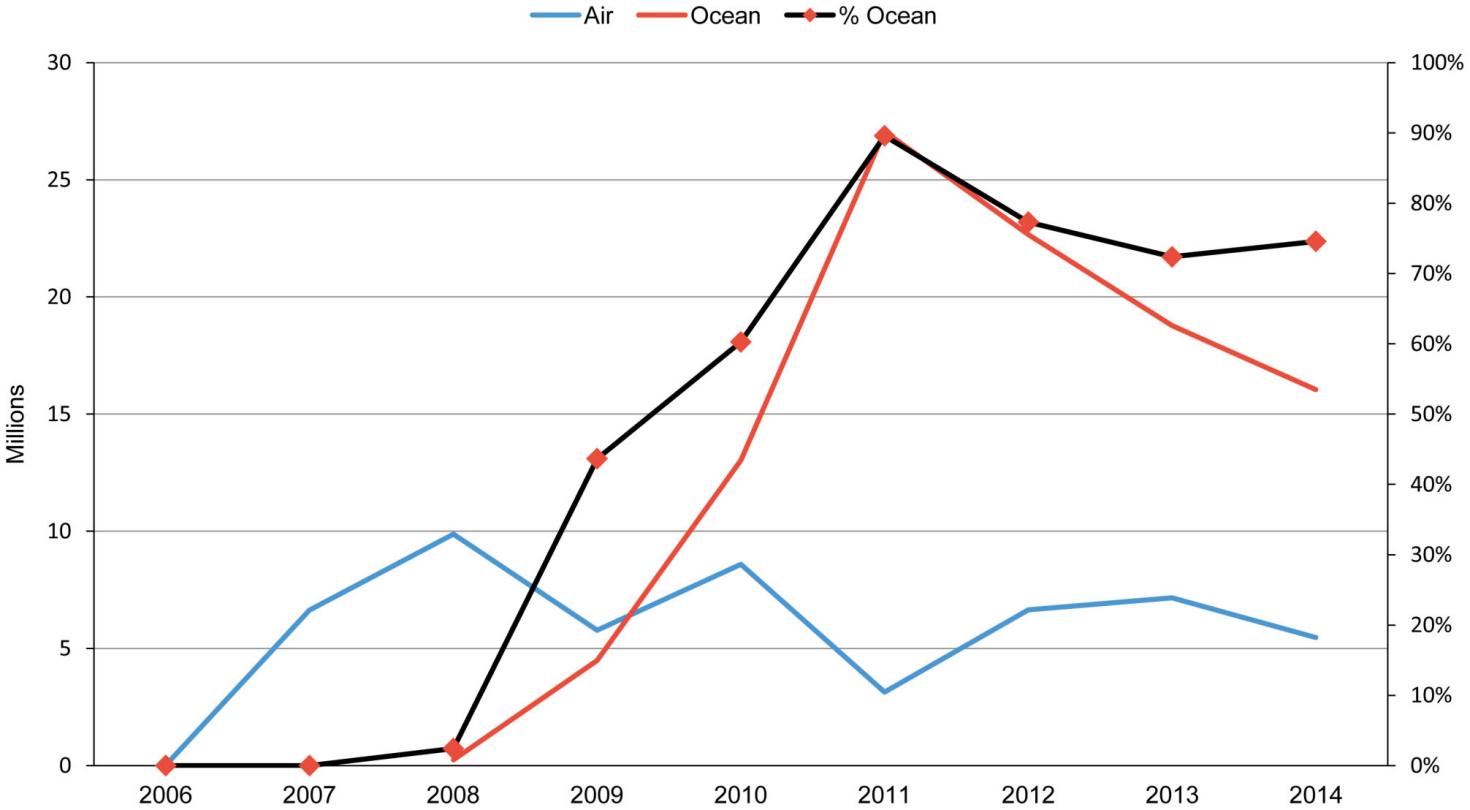
Number of Bottles of ARVs Transported by Air and Ocean and Proportion of Ocean Shipments, 2006–2014 Abbreviation: ARV, antiretroviral.

As shown in Figure 4, the proportion of ARV shipments transported via ocean to country programs increased from less than 10% in 2007 and 2008 (before we fully implemented the transport strategy) to over 75% between 2011 and 2014.^6^ The program estimates that between January 2007 and September 2011, USAID saved US$34 million by leveraging lower-cost transportation to meet client countries' ARV needs.^6^

Between 2007 and 2011, the project saved an estimated US$34 million by leveraging lower-cost transportation.

## CONCLUSION

Through the use of supply chain risk mitigation strategies, particularly by pooling procurement and using regional distribution centers, the program has reduced ARV demand, supply, and cost risks. Among other key impacts, these strategies have resulted in better central-level stock management in PEPFAR-supported countries, improved on-time delivery, and increased cost savings through ocean freight. There are other challenges affecting the in-country ARV supply chain related to human resource capacity, in-country distribution of commodities to treatment sites, reporting and ordering, and storage and infrastructure capacity, which international partners and host countries are addressing collaboratively.

## Notes

**Peer Reviewed**
